# HIV Diagnoses, Prevalence and Outcomes in Nine Southern States

**DOI:** 10.1007/s10900-014-9979-7

**Published:** 2014-12-19

**Authors:** Susan Reif, Brian Wells Pence, Irene Hall, Xiaohong Hu, Kathryn Whetten, Elena Wilson

**Affiliations:** 1 Center for Health Policy and Inequalities Research, Duke University, 310 Trent Drive, Durham, NC 27708 USA; 2 Department of Epidemiology, University of North Carolina School of Public Health, 135 Dauer Drive, Chapel Hill, NC 27599-2089 USA; 3 HIV Incidence and Case Surveillance Branch, Division of HIV/AIDS Prevention, National Center for HIV/AIDS, Viral Hepatitis, STD, and TB Prevention, 1600 Clifton Road, MS E 47, Atlanta, GA 30333 USA

**Keywords:** HIV, AIDS, Southern United States, Mortality, HIV diagnosis

## Abstract

A group of nine states in the Southern United States, hereafter referred to as the targeted states, has experienced particularly high HIV diagnosis and case fatality rates. To provide additional information about the HIV burden in this region, we used CDC HIV surveillance data to examine characteristics of individuals diagnosed with HIV in the targeted states (2011), 5-year HIV and AIDS survival, and deaths among persons living with HIV (2010). We used multivariable analyses to explore the influence of residing in the targeted states at diagnosis on deaths among persons living with HIV after adjustment for demographics and transmission risk. In 2011, the targeted states had a higher HIV diagnosis rate (24.5/100,000 population) than the US overall (18.0/100,000) and higher proportions than other regions of individuals diagnosed with HIV who were black, female, younger, and living in suburban and rural areas. Furthermore, the targeted states had lower HIV and AIDS survival proportions (0.85, 0.73, respectively) than the US overall (0.86, 0.77, respectively) and the highest death rate among persons living with HIV of any US region. Regional differences in demographics and transmission risk did not explain the higher death rate among persons living with HIV in the targeted states indicating that other factors contribute to this disparity. Differences in characteristics and outcomes of individuals with HIV in the targeted states are critical to consider when creating strategies to address HIV in the region, as are other factors identified in previous research to be prominent in the region including poverty and stigma.

## Background

The Southern United States (US) has been consistently identified as being disproportionately affected by HIV. In 2010, persons living in the South had the highest HIV and AIDS diagnosis rates and the highest number of individuals living with HIV of all US regions [1, 2]. Although only 37 % of the US population resides in the South, nearly half (49 %) of individuals living with HIV in 2010 were diagnosed in the South [3]. Persons living with HIV who were diagnosed in the South also had the lowest 3-year HIV survival rates (2002–2006) according to a Centers for Disease Control (CDC)-authored publication [2]. The authors of this publication argued that focus on this area of high HIV burden is warranted to successfully address HIV disease.

A group of Southern states are disproportionally affected by HIV and share certain characteristics, such as overall poor health of the population, high poverty rates, and negative health outcomes for those infected with HIV [4]. For the purpose of this article, these states are referred to as the “targeted states” and include Alabama, Florida, Georgia, Louisiana, Mississippi, North Carolina, South Carolina, Tennessee and Texas. These states have also been referred to as belonging to the “Deep South” [5]. The Deep South has been defined as a region whose states have a shared history of active promotion of slavery and a strong agricultural and economic base in cotton and tobacco [6]. The targeted states have higher levels of STDs and individuals without health insurance than other US regions, including the rest of the Southern states [4, 7–10] as well as high levels of HIV-related stigma [11]. These factors are implicated in negatively influencing HIV transmission and outcomes [4, 12–16].

When grouped as a region, the targeted states have the highest HIV diagnosis rates of any US region, including the rest of the Southern states (2008–2011) [7, 10]. In addition, previous research has found HIV case fatality rates, which are the number of deaths due to HIV among individuals living with HIV in a given year, to be high in the targeted states region, as eight of ten states with the highest HIV case fatality rates from 2002 to 2006 were targeted states [17].

These HIV epidemiologic data from the targeted states suggest a need for increased efforts to address HIV in this region. To create strategies for effective intervention, more detailed information regarding the characteristics and health outcomes of HIV-infected individuals in the targeted states region is needed. This manuscript addresses this information gap, reporting on HIV surveillance data to examine the demographic and HIV risk characteristics of individuals diagnosed and living with HIV in the targeted states region and to compare these to characteristics of persons diagnosed and living with HIV in other US regions. In addition, we examined HIV and AIDS survival and deaths among persons living with HIV in the targeted states region and compared these rates to those in other US regions. Finally, we examined the influence of residing in the targeted states region at HIV diagnosis on deaths among persons living with HIV after adjustment for individual characteristics including demographics and transmission risk category.

## Methods

Data from the Center for Disease Control and Prevention’s (CDC) National HIV Surveillance System were used to examine rates of HIV diagnosis, prevalence and survival among persons diagnosed with HIV [1]. All US states and the District of Columbia report information on persons diagnosed with HIV infection to the CDC in a uniform format and without identifying information.

We analyzed data on adults and adolescents (aged 13 years and older) diagnosed with HIV infection regardless of stage of disease at diagnosis through December 2011, and reported to the CDC through June 2012. We determined the number and rate of persons living with a diagnosis of HIV infection at the end of December 2010 to allow sufficient time for reporting of deaths. Stage of disease at diagnosis was classified according to the reported CD4 T-lymphocyte count or percentage, or documentation of an AIDS-defining condition at or within 3 months of HIV diagnosis.

Persons were assigned to states based on residence at diagnosis. The US regions were defined using the US Census definition. These include Northeast (Connecticut, Maine, Massachusetts, New Hampshire, New Jersey, New York, Pennsylvania, Vermont, and Rhode Island); Midwest (Illinois, Indiana, Iowa, Kansas, Michigan, Minnesota, Missouri, Nebraska, North Dakota, Ohio, South Dakota, and Wisconsin); South (Alabama, Arkansas, Delaware, Florida, Georgia, Kentucky, Louisiana, Maryland, Mississippi, North Carolina, Oklahoma, South Carolina, Tennessee, Texas, Virginia, and West Virginia); and West (Alaska, Arizona, California, Colorado, Hawaii, Idaho, Montana, Nevada, New Mexico, Oregon, Utah, Washington, and Wyoming). Targeted states include Alabama, Florida, Georgia, Louisiana, Mississippi, North Carolina, South Carolina, Tennessee and Texas.

Transmission categories were based on CDC’s hierarchical classification system. [male-to-male sexual contact (men who have sex with men, MSM); injection drug use; MSM who also inject drugs; heterosexual contact with a person known to have or be at high risk for HIV infection; and other] [1]. We used the standard definition of Metropolitan Statistical Area (MSA) category at diagnosis as either urban (metropolitan area >500,000 population), suburban (metropolitan area of 50,000–499,999 population) or rural (nonmetropolitan population) [18].

HIV diagnosis and prevalence rates per 100,000 population were calculated using official estimates from the US Census Bureau [19]. In addition, HIV diagnosis and prevalence rates in the targeted states were stratified by race/ethnicity, age, sex, and MSA category (rural, suburban, and urban). Population denominators were not available from the US Census or other data sources to determine rates by transmission category so only proportions are included [1]. Analyses were adjusted for delays in reporting diagnoses and deaths and for missing risk factor information, but not for incomplete reporting [1].

We determined 5-year survival probabilities among persons diagnosed with HIV during 2003–2004 with Kaplan–Meier survival analyses [20]. A similar methodology was used to examine 5-year survival after an AIDS diagnosis in 2003–2004.

Death rates among persons living with HIV for 2010 were calculated as the number of deaths per 1,000 persons living with HIV. Rate ratios and confidence intervals were calculated to examine differences in deaths among persons living with HIV by sex, race/ethnicity, MSA category, transmission category and geographic region at diagnosis. In addition, adjusted death rates among persons living with HIV and rate ratios were estimated to examine geographical differences in deaths among persons living with HIV after adjustment for regional differences in the distribution of sex, race/ethnicity, transmission category, age, and MSA category.

## Results

### HIV Diagnoses (2011) in the Targeted States (Table 1)

In 2011, 17,732 persons were diagnosed with HIV in the targeted states, representing 38 % of those diagnosed with HIV in the United States (Table 1). Nearly one-quarter (23 %) were female and a majority, 57 %, were black/African American (hereafter referred to as black) The highest percentage of new diagnoses in the targeted states region, 27 %, was among persons aged 25–34 years; however, nearly one-quarter, 23 %, were aged 13–24 years. The percentages of HIV diagnoses occurring among women, blacks, and individuals aged 13–24 were higher in the targeted states than the US average (Table 1).

**Table 1:** Characteristics of individuals diagnosed with HIV in selected states in the Southern United States^a^ and US regions, 2011

	Targeted States		United States		South		South non-targeted states		Northeast		Midwest		West	
	%	Rate	%	Rate	%	Rate	%	Rate	%	Rate	%	Rate	%	Rate
Sex														
Male	76.7	38.7	79.1	29.2	76.5	36.9	75.7	31.5	75.9	31.9	80.8	17.9	88.3	25.5
Female	23.3	11.1	20.9	7.4	23.5	10.7	24.3	9.6	24.1	9.4	19.2	4.1	11.7	3.3
Race/ethnicity														
Black/African American	56.5	72.9	46.6	70	58	73.7	63.6	76.5	43.9	81.8	48.6	53	18.3	57.8
Hispanic/Latino	17.6	24.5	20.9	24.9	15.7	24.8	8.3	27.2	26	44.1	10.9	19	35.9	19.5
White	23	9.5	28	7.7	23.3	8.8	24.3	7	25.6	7.4	35.6	4.8	37.4	9.7
Multiple races	1.7	41.1	2	25.2	1.8	35.2	2.1	24.7	2.2	41.5	3	28	1.5	9.3
Other	1.2	9.1	2.6	8.1	1.3	8.3	1.6	6.7	2.3	7.7	1.9	6.5	6.8	8.5
Age at diagnosis														
13–24	23.3	28.2	21.4	19.2	23	26.9	22.2	22.8	18.8	19.6	25.4	13.7	17.2	11.9
25–34	27.1	40.9	27.9	31.1	26.9	38.9	26.2	32.7	27.1	35.9	28.2	19.5	31	25.7
35–44	21.3	32.6	22.4	25.7	21.2	31.3	21.1	27.3	23.3	30.5	21.6	15.5	24.8	22
45–54	19	27.4	19.2	20	19.3	26.5	20.5	23.7	20.3	22.8	17.5	10.8	18.8	16.1
55+	9.4	7.6	9.2	5.4	9.5	7.3	10	6.5	10.5	6.7	7.2	2.5	8.1	4
*Transmission category*														
Male														
Male-to-male sexual contact	78.1	NA	79	NA	77.6	NA	75.8	NA	72.2	NA	83.9	NA	85.5	NA
Injection drug use	4.3	NA	5.6	NA	4.6	NA	5.6	NA	9.8	NA	4.2	NA	4.6	NA
Male-to-male sexual contact and injection drug use	3	NA	3.6	NA	3	NA	3.1	NA	2.9	NA	3.7	NA	5.7	NA
Heterosexual contact	14.5	NA	11.7	NA	14.7	NA	15.3	NA	15	NA	7.9	NA	4.2	NA
Other^b^	0.1	NA	0.1	NA	0.1	NA	0.2	NA	0	NA	0.2	NA	0.1	NA
Female														
Injection drug use	11.5	NA	14.1	NA	11.6	NA	12.1	NA	16.9	NA	14.4	NA	20.5	NA
Heterosexual contact	88.3	NA	85.7	NA	88.2	NA	87.8	NA	83.1	NA	85.3	NA	79	NA
Other^b^	0.1	NA	0.2	NA	0.1	NA	0.1	NA	0.1	NA	0.3	NA	0.5	NA
Residence at diagnosis														
Urban (MSAs with population ≥500,000)	72.2	29.6	81.7	22.5	74.7	29.5	83.7	29.4	92.5	23.4	80.3	15.7	89.2	17.4
Suburban (metropolitan areas with population 50,000–499,999)	16.5	18.6	11.1	11.1	14.7	16.5	7.9	8.8	4.8	9.4	12.5	6.2	7.9	6.8
Rural (nonmetropolitan areas)	10.7	14.4	6.6	7.3	10	11.9	7.2	6.1	2.2	4.5	6.8	3.2	2.5	3.6
Unknown	0.5	–	0.5	–	0.6	–	1.1	–	0.5	–	0.4	–	0.4	–
Stage of disease at diagnosis														
Stage 3 (AIDS)^c^	28.5	NA	28.7	NA	28.3	NA	27.8	NA	29.2	NA	28.4	NA	29.2	NA
Stage 1 or 2 or unknown	71.5	NA	71.3	NA	71.7	NA	72.2	NA	70.8	NA	71.6	NA	70.8	NA
Total	100	24.5	100	18	100	23.5	100	20.2	100	20.2	100	10.8	100	14.3

^a^Includes Alabama, Florida, Georgia, Louisiana, Mississippi, North Carolina, South Carolina, Tennessee, Texas

^b^Includes hemophilia, blood transfusion, perinatal exposure, and risk factor not reported or not identified

^c^AIDS within 3 months of HIV diagnosis

The HIV diagnosis rate among blacks in the targeted states (72.9 per 100,000) was higher than the overall US rate (70.0) but somewhat lower than the rest of the South (76.5) and the Northeast (81.8). The diagnosis rate among Hispanics or Latinos in the targeted states region (24.5) was comparable to the US rate (24.9). For individuals aged 13–24, the targeted states had a higher HIV diagnosis rate (28.2/100,000) than the overall US rate (19.2/100,000) and the targeted state HIV diagnosis rate among this age group was more than double the rate in the West and Midwest. The HIV diagnosis rate was also the highest in the targeted states region for all other age ranges when compared to other regions.

A lower percentage of persons diagnosed with HIV in 2011 in the targeted states resided in urban areas (72 %) compared with the United States overall (82 %). In the targeted states region, 17 % of persons diagnosed with HIV resided in suburban areas and 11 % in rural areas compared to 11 and 7 % of persons diagnosed in the entire United States, respectively. In the rest of the South, only 8 % of persons diagnosed with HIV lived in suburban areas and 7 % in rural areas. Although a lower percentage of HIV diagnoses in the targeted Southern states were among individuals living in urban areas compared to the United States overall, the targeted states had a higher HIV diagnosis rate (29.6/100,000) in urban areas when compared to the United States overall and the Northeast region (22.5 and 23.4 respectively). The targeted states region also had a higher HIV diagnosis rate in suburban areas (18.6/100,000) and rural areas (14.4/100,000) compared to the United States overall (11.1 and 7.3 respectively).

Among both men and women, the targeted states had a higher percentage of diagnoses that were attributed to heterosexual contact (14.5 and 88.3 % respectively) and a lower percentage attributed to injection drug use (IDU) (4.3 and 11.5 % respectively) when compared to the United States overall for heterosexual contact (11.7 and 85.7 % respectively) and IDU (5.6 and 14.1 % respectively).

### HIV Prevalence, Year End 2010 (Table 2)

In 2010, there were an estimated 285,677 persons living with HIV in the targeted states; the highest number of any US region. As with HIV diagnosis, the targeted states had a higher percentage of individuals living with HIV that were female (27.7 %) than the overall United States (24.9 %) (Table 2). The targeted states also had a higher percentage of individuals living with HIV who were black (54.2 %) than the United States overall (43.6 %) but less than the rest of the South (63.2 %). In addition, the targeted states had a lower percentage of males and females living with HIV with a transmission category of IDU and a greater percentage with heterosexual contact as the reported transmission category than the United States overall and the rest of the Southern states. Over one-quarter of individuals living with HIV in the targeted states were living in suburban or rural areas (15 % suburban and 11 % rural) at the time of diagnosis, which was the largest percentage of individuals living with HIV that had been diagnosed outside of urban areas in any region.

**Table 2:** HIV prevalence in selected Southern States^a^ and other United States Regions, year-end 2010

	Targeted states		United States		South		Southern non-target		Northeast		Midwest		West	
	%	Rate	%	Rate	%	Rate	%	Rate	%	Rate	%	Rate	%	Rate
Sex														
Male	72.3	595.9	75.1	521	72	575.7	71	514	69.5	717.6	78.9	294.8	87.4	493.2
Female	27.7	215.4	24.9	164.4	28.0	211.1	29	198.2	30.5	291.7	21.1	75.0	12.6	70.0
Race/ethnicity														
Black/African American	54.2	1,144.9	43.6	1,231.6	56.2	1,184.9	63.2	1,321.8	42.9	1,966.7	44.2	815.5	16.2	1,001.0
Hispanic/Latino	14.6	336.9	19.0	433.0	12.6	336.5	5.7	332.4	27.1	1,148.4	9.5	288.9	27.7	297.0
White	29.0	194.7	33.8	173.1	28.8	179.3	28.1	139.9	26.0	183	42.5	97.1	50.6	254.1
Multiple races	1.5	622.1	1.9	474.2	1.6	536.6	1.9	386.5	2.9	1,334.5	2.3	367.5	1.2	152.0
Other	0.7	85.4	1.6	97.6	0.8	85.0	1.2	84.3	1.2	102.3	1.5	85.0	4.3	105.1
Age at end of year														
13–24	5.2	101.3	4.5	74.4	5.1	97.8	4.9	86.8	4.2	106.5	5.4	48.6	2.9	38.1
25–34	16.5	407.5	14	295.1	16.1	385.9	14.6	319.8	10.9	359.9	15.6	183	12.8	208.2
35–44	28.1	692.0	26.7	565.1	27.8	665.9	26.4	584.8	24.3	765.7	28.1	332.4	26.8	458.2
45–54	33.6	780.5	35.5	685	34	759.6	35.3	697.9	37.5	1,026.9	34.2	348.5	36.9	607.1
55+	16.6	222.3	19.3	216.7	17.1	220.8	18.7	216.2	23.0	363.1	16.7	98.5	20.6	202.7
*Transmission category*														
Male														
Male-to-male sexual contact	68.4	NA	67.7	NA	67.2	NA	63.0	NA	55.1	NA	75.2	NA	78.4	NA
Injection drug use	9.3	NA	13.0	NA	10.6	NA	15.5	NA	25.0	NA	8.6	NA	6.4	NA
Male-to-male sexual contact and injection drug use	7.0	NA	7.5	NA	7.0	NA	6.9	NA	5.7	NA	7.7	NA	10.5	NA
Heterosexual contact	14.5	NA	10.8	NA	14.3	NA	13.6	NA	12.6	NA	7.4	NA	4.0	NA
Other^b^	0.9	NA	1.1	NA	0.9	NA	1.1	NA	1.6	NA	1.1	NA	0.6	NA
Female														
Injection drug use	18.0	NA	24.9	NA	19.7	NA	25.5	NA	32.6	NA	21.9	NA	26.8	NA
Heterosexual contact	79.8	NA	72.3	NA	78	NA	72.2	NA	63.9	NA	75.5	NA	69.6	NA
Other^b^	2.2	NA	2.8	NA	2.2	NA	2.2	NA	3.5	NA	2.6	NA	3.6	NA
Residence at diagnosis														
Urban (MSAs with population ≥500,000)	73.6	493.9	82.8	428.8	75.6	496.7	82.6	505.7	91.4	568.1	79.1	261.1	89.2	340.0
Suburban (metropolitan areas with population 50,000–499,999)	14.9	271.3	10.0	185.6	13.5	248.2	8.6	164.4	4.9	236.7	13.0	109.2	7.5	126.9
Rural (nonmetropolitan areas)	10.6	231.6	6.2	127.7	9.8	192.2	6.9	101.0	2.7	137.7	6.8	53.5	2.7	75.9
Unknown	0.9	–	1.0	–	1.1	–	1.8	–	1.0	–	1.1	–	0.6	–
Total	100	400.3	100	338.3	100	388.2	100	351.5	100	496.6	100	182.3	100	279.8

^a^Includes Alabama, Florida, Georgia, Louisiana, Mississippi, North Carolina, South Carolina, Tennessee, Texas

^b^Includes hemophilia, blood transfusion, perinatal exposure, and risk factor not reported or not identified

The HIV prevalence rates for both men (595.9/100,000) and women (215.4/100,000) were higher in the targeted states than the US average among men (521.0/100,000) and women (164.4/100,000) and all other regions except for the Northeast (717.6 for men and 291.7 for women). For the age ranges 35–44, 45–54, and over 55, the targeted states had prevalence rates higher than the US average but substantially lower than the Northeast. However, the HIV prevalence rate among 25–34 year olds was higher in the targeted states (407.5 per 100,000) than the Northeast (359.9 per 100,000) and the prevalence rate among 13–24 year olds was comparable between the targeted states (101.3) and Northeast (106.5). Finally the HIV prevalence rate in rural and suburban areas was higher in the targeted states in comparison to the Northeast and all other regions.

### Survival Among Persons Diagnosed with HIV

The 5-year survival in the targeted states was equal for men and women and decreased with age (Table 3). Blacks had lower survival proportions at 5 years (0.84) than whites (0.87) in the targeted states. Reporting injection drug (IDU) use as transmission risk was associated with a substantially lower 5-year survival proportion (0.80) than heterosexual contact (0.88) among females. Among males, IDU as a transmission category was also associated with lower 5-year survival (0.80) than MSM (0.90) and heterosexual contact (0.83). Five-year survival among those diagnosed in rural areas of the targeted states (0.82) was lower than among those diagnosed in urban areas of the targeted states (0.86).

**Table 3:** Survival for more than 60 months after a diagnosis of AIDS or a diagnosis of HIV infection, adults and adolescents (aged 13 years or over) diagnosed in 2003–2004 in selected Southern States^a^ and other United States Regions

	Proportion survived	
	AIDS survival	HIV survival
United States^b^	0.77	0.86
Targeted states^b^	0.73	0.85
State of residence		
Alabama	0.69	0.86
Florida	0.72	0.85
Georgia	0.75	0.86
Louisiana	0.67	0.81
Mississippi	0.68	0.83
North Carolina	0.74	0.85
South Carolina	0.73	0.84
Tennessee	0.72	0.85
Texas	0.76	0.87
*Targeted states* ^*b*^		
Sex		
Male	0.74	0.85
Female	0.71	0.85
Race/ethnicity		
Black/African American	0.70	0.84
Hispanic/Latino	0.78	0.87
White	0.76	0.87
Multiple races	0.73	0.84
Other	0.78	0.89
Age at diagnosis		
13–24	0.83	0.95
25–34	0.79	0.91
35–44	0.75	0.86
45–54	0.67	0.78
55+	0.52	0.61
*Transmission category*		
Male adult or adolescent		
Male-to-male sexual contact	0.79	0.90
Injection drug use	0.66	0.80
Male-to-male sexual contact and injection drug use	0.75	0.88
Heterosexual contact	0.72	0.83
Other^c^	0.64	0.76
Female adult or adolescent		
Injection drug use	0.63	0.80
Heterosexual contact	0.75	0.88
Other^c^	0.66	0.82
Residence at diagnosis		
Urban (MSAs with population ≥500,000)	0.73	0.86
Suburban (metropolitan areas with population 50,000–499,999)	0.71	0.84
Rural (nonmetropolitan areas)	0.71	0.82
Unknown	0.82	0.93

^a^Includes Alabama, Florida, Georgia, Louisiana, Mississippi, North Carolina, South Carolina, Tennessee, Texas

^b^Excludes cases diagnosed prior to the earlier date of HIV code-based and name-based reporting dates. Therefore, (1) ‘United States’ excludes PA, (2) ‘Targeted states’ includes all the nine targeted-states (GA has only 2004 data), and (3) ‘Other states’ includes the other states (40 states + DC excluding PA) of (1)

^c^Includes hemophilia, blood transfusion, perinatal exposure, and risk factor not reported or not identified

In the targeted states, the 5-year survival proportion after HIV diagnosis (for diagnoses in 2003–2004) was lower (0.85) compared with the non-targeted states (0.87). There were differences in survival proportions between the targeted states. Texas had the highest 5-year HIV survival proportion (0.87), which was the same as the survival proportion for the non-targeted states. Louisiana had the lowest 5-year survival proportion (0.81) followed by Mississippi (0.83) and South Carolina (0.84).

### Survival Among Persons Ever Diagnosed with AIDS

In the targeted states, females had a lower 5-year AIDS survival proportion (0.71) than males (0.74) (Table 3). Blacks had the lowest survival proportion (0.70) of any race/ethnicity and survival decreased with age. For example, in the targeted states among those diagnosed with AIDS who were 55 or older, 48 % died within 5 years of AIDS diagnosis whereas 17 % of those 18–24 died within 5 years of AIDS diagnosis. The 5-year survival proportion after an AIDS diagnosis was lower in suburban and rural areas (0.71) than in urban areas (0.73).

For 5-year survival after an AIDS diagnosis, the targeted states had the lowest survival proportion of any region (0.73), indicating that 27 % of those diagnosed with AIDS in 2003–2004 had died within 5 years. The 5-year AIDS survival proportion was higher for the entire United States (0.77), the Northeast (0.79), the Midwest (0.78) and the West (0.82). Similar to HIV survival, there was variation in 5-year AIDS survival in the targeted states with Louisiana having the lowest survival rate at 0.67, indicating that one-third of individuals diagnosed with AIDS in Louisiana in 2003–2004 had died within 5 years of diagnosis. The next lowest survival proportions in the targeted states were Mississippi (0.68) and Alabama (0.69). No targeted state had a 5-year AIDS survival proportion at or above the overall US survival proportion.

### Deaths Among Persons Living with HIV

The death rate among persons living with HIV was higher in the targeted states (27.3 per 1,000 persons estimated to be living with HIV) than in any other region (non-targeted Southern states: 24.6; Northeast 24.7, Midwest 20.7, West 18.8.) (Table 4). After adjustment for age, sex, transmission category, and area population size, these differences were accentuated or substantively unchanged and the death rate among persons living with HIV in the targeted states was significantly higher than in the other regions, e.g., adjusted rate ratio targeted states versus non-targeted Southern states [Rate Ratio 0.83; 95 % confidence intervals (CI) 0.79, 0.87].

**Table 4:** Death rate among persons living with HIV, adjusted and adjusted by United States region and by specific characteristics within selected Southern States^a^, 2010

	Unadjusted rate	Adjusted rate^d^	Rate ratio (CI)
	Rate of deaths 2010 (among 1,000 PLWH and new diagnoses during 2010)^c^	Rate of deaths 2010 (among 1,000 PLWH and new diagnoses during 2010)^c^	
United States	24.0		
Region of residence			
Northeast	24.7	22.3	0.77 (0.74,0.80)
Midwest	20.7	22.5	0.77 (0.74,0.81)
South	26.7	28.0	
Targeted states^a^	27.3	29.0	Reference
South non-targeted states	24.6	24.0	0.83 (0.79,0.87)
West	18.8	21.2	0.73 (0.70,0.76)
State of residence			
Alabama	30.7		
Florida	29.1		
Georgia	28.4		
Louisiana	34.5		
Mississippi	28.6		
North Carolina	25.6		
South Carolina	29.6		
Tennessee	25.0		
Texas	22.2		
*Targeted states*			
Sex			
Male	26.9		
Female	28.2		
Race/ethnicity			
Black/African American	30.0		
Hispanic/Latino	19.8		
White	25.5		
Multiple races	41.1		
Other	12.4		
Age category			
13–24	7.5		
25–34	13.5		
35–44	20.3		
45–54	31.2		
55+	56.9		
*Transmission category*			
Male adult or adolescent			
Male-to-male sexual contact	21.5		
Injection drug use	45.1		
Male-to-male sexual contact and injection drug use	36.1		
Heterosexual contact	36.0		
Other^b^	26.3		
Female adult or adolescent			
Injection drug use	39.0		
Heterosexual contact	26.0		
Other^b^	18.0		
Residence at diagnosis			
Urban (MSAs with population ≥500,000)	25.6		
Suburban (metropolitan areas with population 50,000–499,999)	32.5		
Rural (nonmetropolitan areas)	31.8		
Unknown	22.4		

^a^Includes Alabama, Florida, Georgia, Louisiana, Mississippi, North Carolina, South Carolina, Tennessee, and Texas

^b^Includes hemophilia, blood transfusion, perinatal exposure, and risk factor not reported or not identified

^c^Rates are per 1,000 persons living with diagnosed HIV infection (PLWH), denominator was calculated as (No. of PLWH at the end of 2009 + new diagnoses during 2010)

^d^Adjusted for age, race/ethnicity, sex, transmission category, residence at diagnosis

Within the targeted states, blacks had a higher death rate among persons living with HIV than whites (rate ratio 1.2, CI 1.12, 1.24; data not shown) and the death rate among persons living with HIV increased with age category. Persons with HIV infection attributed to injection drug use or heterosexual contact had higher death rates among persons living with HIV than persons with infection attributed to male-to-male sexual contact (1.97, CI 1.86, 2.10 and 1.36, CI 1.29, 1.43 respectively). Suburban and rural residence at diagnosis significantly predicted greater death rates among persons living with HIV compared to urban residence at diagnosis (1.27, CI 1.12 1.34 and 1.24, CI 1.16 1.33, respectively). Louisiana had the highest death rate among persons living with HIV and Texas had the lowest death rate among persons living with HIV of all targeted states.

## Discussion

Our results indicate that in 2011 the targeted states had the highest HIV diagnosis rate of any US region. The percentage of individuals diagnosed with HIV in the targeted states was disproportionate to the population size, as 38 % of individuals diagnosed with HIV resided in the targeted states, while the targeted states accounted for 28 % of the US population [1, 3]. Persons diagnosed with HIV in the targeted states region reflected higher proportions of women, blacks, and individuals residing in suburban and rural areas than the overall United States.

In the targeted states, a higher proportion of persons diagnosed with HIV were adolescents and young adults than in the United States overall and HIV diagnosis rates among individuals 13–24 and 25–34 were higher than in other US regions. Higher HIV diagnosis rates among the younger age categories, particularly in the targeted states region and the rest of the South, could be attributed, in part, to lack of education about HIV transmission and less gravity placed on HIV infection due to improvements in available drug regimens [21]. The higher concentration of HIV in younger ages in the targeted states is an important factor to consider in prevention and treatment planning, as response to prevention education and interventions may differ by age [22]. Development and implementation of effective prevention and treatment strategies for the younger population will be critical to stemming HIV transmission in the targeted Southern states.

The targeted states had higher HIV diagnosis rates than all other regions in urban areas, as well in suburban and rural areas indicating that the targeted states region is grappling with significant and disproportionate HIV burden in both urban and more rural areas. Challenges to HIV prevention and care in rural and suburban areas of the targeted states, such as lack of transportation, lack of qualified providers, and HIV-related stigma [23–27], may provide some explanation for the study findings regarding lower 5-year HIV and AIDS survival proportions in the targeted states in comparison to other US regions. In addition, the death rate among persons living with HIV (2010) was found to be higher in the targeted states compared to the overall United States; with rural and suburban areas having higher death rates among persons living with HIV than urban areas of the targeted states. The higher death rate among persons living with HIV in the targeted states suggests a disconnect between diagnosis and maintenance of HIV care in this region, particularly in non-urban areas. Identifying effective ways to structure prevention and care services so that they address common barriers to care such as accessibility and pervasive stigma will be critical to improving HIV outcomes in rural and suburban areas of the targeted states [4, 11, 24, 26].

Regional differences in the characteristics of individuals living with HIV, including sex, race/ethnicity, mode of transmission, MSA category and age, did not explain the higher death rate among persons living with HIV in the targeted states compared with other US regions. Rather, the disparity in the death rate among persons living with HIV between the targeted states and other regions were substantively unchanged or accentuated after adjustment, suggesting that additional factors unmeasured in the data contribute to the greater risk of death among persons living with HIV in the targeted states. These contributing factors likely include characteristics of the targeted states such as lower levels of income, education, and insurance coverage and higher levels of HIV stigma and racism [4, 9, 11, 28, 29]. Previous research has consistently related HIV-related stigma to negative outcomes, including poor medication adherence and greater HIV risk behavior [12–16]. A recent qualitative study among young black MSM reported that HIV-related stigma and homophobia were related to sexual risk behavior, reluctance to obtain HIV testing or care, and poorer medication adherence [30]. An additional contributing factor may be the social class system unique to the US South that has traditionally allowed for little social mobility, along with marginalization of, and discrimination against certain groups and often resulting in distrust of systems of care among those in a lower social strata [31–33]. These societal factors have likely collectively contributed to creating an environment in the targeted states in which HIV infection is more likely and health outcomes for HIV-positive individuals are poorer than in other US regions. Additional research is needed to better determine and understand the factors that influence the higher death rate among persons living with HIV in the targeted states and to identify effective mechanisms to address known barriers including HIV stigma.

Among the targeted states, HIV survival and deaths among persons living with HIV were not uniform. For example, Texas had a 5-year AIDS survival proportion only slightly lower than the overall US survival proportion, while Louisiana had a substantially lower 5-year survival proportion. The study findings regarding deaths among persons living with HIV are consistent with an analysis by Hanna and Colleagues from an earlier period, 2001–2007, that found all targeted states but Texas to be in the ten states with the highest HIV case fatality rates in the United States [17]. The targeted states with the most concerning mortality statistics, particularly Louisiana, may especially be in need of focused attention on addressing the factors contributing to these concerning statistics.

The findings of this study must be considered in the context of the study limitations. The data were adjusted for delays in reporting, however, they were not adjusted for incomplete reporting, and may slightly underrepresent the actual number of HIV diagnoses in the time period of study. In addition, a portion of deaths may not be reported to the HIV surveillance system [1], affecting the estimate of the number of people living with HIV disease. Relocation is not always accounted for within the surveillance data. Furthermore, the data are presented according to the area of residence at diagnosis and may not reflect the current residence for persons living with a diagnosis of HIV infection. If these potential errors occur more frequently in specific geographical areas rather than occurring randomly throughout the United States they may affect the accuracy of regional comparisons. Finally, the HIV diagnosis data cannot accurately provide information on HIV incidence, as they reflect only diagnoses made rather than occurrence of new HIV infections.

In conclusion, HIV surveillance data indicate a disproportionate impact of HIV in the targeted Southern states in terms of higher HIV diagnosis and prevalence rates and greater HIV mortality. These findings signal a need for effective strategies to address HIV in this region. The characteristics of individuals diagnosed with HIV in the targeted state region differ from the overall United States, as a greater proportion of these individuals in the targeted states are black, female, and younger. The targeted states also have a higher proportion of those diagnosed and living with HIV in suburban and rural areas than any other US region. These differences are crucial to consider, as are other factors prominent in the targeted states including poverty and stigma [4, 34], when creating strategies to address HIV in this region.
